# Bioinformatics based exploration of the anti-liver fibrosis mechanism of Pien Tze Huang via EGFR/JAK1/STAT3 pathway

**DOI:** 10.3389/fimmu.2025.1665648

**Published:** 2025-10-17

**Authors:** Xiaoting Hong, Yonghua Ye, Chunfeng Lin, Miaolan Zheng, Fan Lin, Qing Xiong, Wei Xu, Huang Li, Yuqin Zhang, Haiyin Zheng

**Affiliations:** ^1^ Institute of Structural Pharmacology & Traditional Chinese Medicine (TCM) Chemical Biology, Fujian Key Laboratory of Chinese Materia Medica, College of Pharmacy, Fujian University of Traditional Chinese Medicine, Fuzhou, China; ^2^ College of Integrative Medicine, Fujian University of Traditional Chinese Medicine, Fuzhou, China

**Keywords:** liver fibrosis, Pien Tze Huang, liver inflammation, EGFR/JAK1/STAT3 signaling pathway, network pharmacology

## Abstract

**Object:**

Liver fibrosis is a key stage in chronic liver disease, but targeted treatments are scarce. Pien Tze Huang (PZH), a traditional Chinese medicine, shows anti-fibrotic potential, though its mechanisms are not fully understood.

**Method:**

A liver fibrosis model was created in C57BL/6 mice using carbon tetrachloride (CCL_4_). PZH’s effects were assessed via liver morphology, serum/liver biomarkers, transaminase levels, and histopathology. PZH’s chemical components were identified through database and literature research. Network pharmacology, molecular docking and molecular dynamics simulation were used to investigate the underlying mechanisms, which were validated *in vivo* and *in vitro* with immunofluorescence and Western blot analyses.

**Results:**

PZH significantly attenuated hepatic transaminase disorders, reduced serum procollagen III/IV, alleviated fibrotic liver histopathology, and suppressed macrophage markers and hepatic inflammation. Through multi-database integration, 24 bioactive compounds were identified in PZH, including ginsenoside Rh2. Further investigation showed that PZH ameliorates liver fibrosis by modulating key targets, including AKT1, EGFR, and STAT3. Molecular docking analysis and molecular dynamics simulation demonstrated a significantly high binding affinity between ginsenoside Rh2 and the target proteins EGFR, JAK1, and STAT3. *In vitro and in vivo* experiments confirmed that PZH and ginsenoside Rh2, reduced RAW264.7 inflammatory mediators, inhibited M1 polarization, and downregulated EGFR/JAK1/STAT3.

**Conclusion:**

The findings reveal that PZH ameliorates liver fibrosis by inhibiting macrophage-mediated inflammation via blockade of the EGFR/JAK1/STAT3 signaling axis, providing a mechanistic foundation for its clinical application.

## 1 Introduction

Hepatic fibrosis is a dysregulation condition due to various chronic liver diseases’ damage and repair response. According to a study by the Global Burden of Disease (GBD), approximately 2 billion people worldwide are affected by chronic liver disease, with 25%-30% progressing to liver fibrosis. By 2020, over 12 million new cases were reported, accounting for about 1.4 million deaths. According to epidemiological reports (1, 2), the prevalence of liver fibrosis ranges from 7% to 12% in China. This is primarily driven by hepatitis B virus infection (45% of cases) and non-alcoholic fatty liver disease (NAFLD) (30% of cases). Without effective intervention, liver fibrosis patients in China are projected to exceed 50 million by 2030, with approximately 15% progressing to decompensated cirrhosis (3). Liver fibrosis is a major global health challenge, making it essential to understand its pathophysiology and develop effective treatments.

Current treatments for liver fibrosis, such as antivirals for viral hepatitis, alcohol abstinence, and metabolic control for NAFLD, can partially reverse early fibrosis, but are ineffective for advanced scarring (4). Approved antifibrotics like pirfenidone and nintedanib inhibit hepatic stellate cell (HSC) activation and suppress transforming growth factor-β (TGF-β) signaling pathway (5, 6), but have limited efficacy due to toxicity, side effects, and single-target limitations. There is an urgent need for more effective liver fibrosis treatments.

Traditional Chinese medicine (TCM) is recognized for its multi-target and multi-pathway therapeutic effects. Emerging evidence underscores the significant role of TCM in the prevention and treatment of liver diseases, including hepatocellular carcinoma and viral hepatitis (7, 8). Pien Tze Huang (PZH), a renowned TCM formulation that has been utilized for over 500 years since its development during the Ming Dynasty (1368–1644 CE) as a remedy for inflammatory and infectious diseases (9). Derived from classical formulas like Huanglian Jiedu Tang, PZH contains natural ingredients such as natural *Calculus bovis*, musk, *Pseudoginseng* root, and Snake gallbladder. Essentially, PZH exerts “heat-clearing, detoxifying, and blood-activating” effects, which align with its clinical applications in liver fibrosis and cancer-associated inflammation. While there, PZH is effective in improving liver function and reducing fibrosis markers in preclinical models (10, 11), its precise molecular mechanisms in anti-fibrotic and anti-tumor activities remain incompletely understood.

Network pharmacological analysis, first proposed by Hopkins in 2007, is a promising research field and an advanced approach to elucidate bioactive substances and the pharmacological mechanisms of Chinese herbal medicines through the construction of drug-target-disease interaction networks. Notably, previous research has shown that network pharmacology is effective in elucidating the pharmacodynamic basis and mechanisms of Chinese herbal medicines, including PZH in the treatment and management of colon cancer and *Calculus bovis* in the treatment of liver cancer (12, 13). Notably, this system-driven paradigm has been pivotal in elucidating the anti-inflammatory and anti-fibrotic actions of PZH.

The epidermal growth factor receptor (EGFR) is a receptor tyrosine kinase present on hepatocytes, hepatic stellate cells, and macrophages. It forms a complex with Janus kinase 1 (JAK1) to phosphorylate STAT3 and promote STAT3 entry into the nucleus, which then regulates fibrogenesis-related genes (14, 15). While the EGFR/JAK1/STAT3 pathway is associated with fibrosis in various organs, its role in liver fibrosis remains unclear.

In this study, we initially evaluated the therapeutic effects of PZH on liver fibrosis through the assessment of biochemical indices, histopathological analysis, and pro-inflammatory macrophage transformation in a carbon tetrachloride (CCL_4_)-induced liver fibrosis mouse model. Additionally, we used bioinformatics approaches to explore the potential mechanisms underlying the therapeutic effects of PZH. Finally, we assessed the predictive pathways (the EGFR/JAK1/STAT3 pathway) through immunoblotting of *in vivo* liver tissues and *in vitro* assays using immunohistochemistry and ELISA techniques. This study aims to provide sufficient evidence for the clinical application of the PZH approach.

## 2 Materials and methods

### 2.1 Chemical and biological reagents

The BCA Protein Assay Kit and BeyoColor™ Prestained Protein Marker (6.5–270 kDa) were obtained from Beyotime Biotechnology (Shanghai, China). The Hematoxylin and Eosin (H&E) Staining Kit, Masson’s Trichrome Staining Kit, and Lipopolysaccharide (LPS) were purchased from Solarbio Science & Technology Co., Ltd (Beijing, China). The Aspartate Aminotransferase (AST/GOT) Assay Kit and Alanine Aminotransferase (ALT/GPT) Assay Kit were purchased from Nanjing Jiancheng Bioengineering Institute (Nanjing, China). The Nitric Oxide (NO) Detection Kit was bought from Beyotime Biotechnology (Shanghai, China). Antibodies for JAK1, IRF5, EGFR, and STAT3 were purchased from Abcam (Cambridge, UK).

### 2.2 Drug preparation

PZH tablets (Lot No. 2203044; National Drug Approval No. Z35020243) were purchased from Zhangzhou Pien Tze Huang Pharmaceutical Co., Ltd (Zhangzhou, China). The tablets were ground into a fine powder using an agate mortar and dissolved in dimethyl sulfoxide (DMSO) to a final concentration of 4 mg/mL. The solution was sonicated for 30 min at 25 °C, filtered through a 0.22 μm sterile membrane, and stored at 4 °C in light-protected vials for subsequent use.

Ginsenoside Rh2 (Lot No. MUST-21032410; purity ≥98% by HPLC) was purchased from Chengdu Mansite Biotechnology Co., Ltd (Chengdu, China). A stock solution (4 mg/mL) was prepared in DMSO, filtered through a 0.22 μm sterile membrane, and stored at 4 °C under light-protected conditions.

### 2.3 Animal experiment

A total of 40 male C57BL/6 mice (6–8 weeks old, weighing 18–22 g) were purchased from Beijing Huafukang Biotechnology Co., Ltd (Beijing, China). The animals were housed under controlled environmental conditions of temperature 23 ± 2 °C and relative humidity of 50 ± 10%, with a 12-h light/dark cycle. The animals were normally fed and watered. All animal experiments were conducted in strict adherence to the Guidelines for Care and Use of Laboratory Animals of Fujian University of Traditional Chinese Medicine (Ethics No.: FJTCM-IACUC-2022019) and ARRIVE 2.0 reporting standards. Additionally, all animals were ethically treated to minimize their suffering.

After one week of adaptive feeding, the mice were randomly assigned into five groups: vehicle control group (VC), model control group (MC), PZH-L group (0.117 g/kg/day), PZH-M group (0.234 g/kg/day), and PZH-H group (0.468 g/kg/day). Liver fibrosis was induced through intraperitoneal injection of 5 mL/kg body weight of CCL_4_/peanut oil (1:9, v/v). All mice were injected with the CCL_4_/peanut oil solution twice a week for 6 weeks, except for the VC group, which received the same volume of saline solution for 6 weeks. The administration groups received intragastric administration of PZH or saline (10 mL/kg/day) for 6 weeks beginning at one day after CCL_4_ injections. The dose of PZH used in this experiment was selected according to Dose translation from animal to human (16). Additionally, all PZH suspensions were prepared in 0.9% NaCl to achieve target concentrations: 0.048 g/mL (high), 0.024 g/mL (medium), and 0.0117 g/mL (low). The body weights of the mice were monitored daily to adjust the dosing volumes, and the mice were maintained on a high-fat diet throughout the study.

At the end of the experiment, all mice were fasted overnight before blood collection from the ocular venous plexus. The blood samples were double centrifuged at 1500 g for 10 min to obtain serum for subsequent biochemical analysis. After blood collection, the abdominal cavity was opened to excise the liver. After photographic imaging, a 1 × 1 × 0.5 cm tissue block was dissected from the largest hepatic lobe. Residual blood and surface debris were eliminated through washing with ice-cold 0.9% saline. Subsequently, the tissue sections were blotted dry with sterile filter paper. For histopathological evaluation, the samples were fixed in 4% paraformaldehyde (PFA) at 4°C for 48 h, followed by paraffin embedding to conduct hematoxylin-eosin(H&E), Masson’s trichrome staining, and immunohistochemical (IHC) analysis. The remaining liver tissues were flash-frozen in liquid nitrogen, wrapped in aluminum foil, and stored at -80 °C for protein extraction and immunoblotting assays.

### 2.4 Histological staining

The liver tissues were harvested, fixed in 4% paraformaldehyde, embedded in paraffin, and sectioned at a thickness of 4 μm. Notably, histological analysis was performed through H&E staining, while Masson’s trichrome staining was used to assess the fibrotic area. Five random fields of Masson-stained sections from each sample were captured using a light microscope.

### 2.5 Measurement of serum liver function markers

Serum levels of aspartate aminotransferase (AST) and alanine aminotransferase (ALT) were quantified using a microplate-based colorimetric assay based on the kit instructions from the manufacturer. Briefly, 10 μL of serum was mixed with 100 μL of active reagent, and absorbance was measured at 510 nm using a microplate reader after incubation at 37 °C for 30 min. The ELISA technique was used to assess the concentrations of type III procollagen (PCIII) and type IV collagen (CIV) in serum. Serum samples (50 μL) were seeded into the pre-coated 96-well plates, followed by incubation with detection antibodies and horseradish peroxidase (HRP)-conjugated streptavidin. Consequently, a stop solution was used to terminate the reaction, and absorbance was measured at 450 nm. Notably, following the instructions on the kit, a standard curve was generated, and the protein concentrations of the samples were then determined accordingly.

### 2.6 Immunohistochemistry and immunofluorescence

Immunohistochemistry and immunofluorescence were performed to detect the protein expression of CD68 in liver tissues in all groups. Liver tissue sections were deparaffinized, hydrated, and incubated with 3% H_2_O_2_ for 5~10 min. They were then blocked with buffer and incubated with primary antibody CD68 at 4 °C overnight. This was followed by incubation with the secondary antibody for 30 min at 37 °C, and then with SABC at 37 °C for 10 min. The 3,3′-Diaminobenzidine tetrahydrochloride (DAB) solution was added to examine the immunoreactivity. The nucleus was stained with hematoxylin, and sections were sealed with neutral gum. Finally, images were captured using an optical microscope (Nikon Corporation, Tokyo, Japan; model ECLIPSE 511).

### 2.7 Bioinformatics analysis

#### 2.7.1 Prediction of prospective PZH targets associated with liver fibrosis

The bioactive compounds of PZH, including *Panax notoginseng, Calculus bovis*, Snake gallbladder, and *Moschus*, were systematically retrieved from the TCMSP (Traditional Chinese Medicine Systems Pharmacology, https://www.tcmsp-e.com/) (17), TCMID (Traditional Chinese Medicine Integrated Database) (18), PubMed (19), and CNKI (China National Knowledge Infrastructure) databases. Potential active ingredients were selected as compounds attaining the oral bioavailability (OB) ≥30% and drug-likeness (DL) ≥0.18. Canonical simplified molecular input line entry system(SMILES) of these components were downloaded from the PubChem database (https://pubchem.ncbi.nlm.nih.gov) and uploaded to the Swiss Target Prediction platform (http://www.swisstargetprediction.ch) to predict protein targets (probability score ≥0.3) (17). Additionally, the target gene names were standardized using the UniProt database (https://www.uniprot.org, release 2024-05). Construction of the drug-component-target network was conducted and then visualized using Cytoscape 3.7.2 (https://cytoscape.org) to elucidate interactions among PZH constituents, bioactive components, and potential targets.

Liver fibrosis-related targets were retrieved from the Genecards (version 5.22.0, https://www.genecards.org) (20) and Online mendelian inheritance in man(OMIM) (https://www.omim.org) (21) databases using the keywords “liver fibrosis” and “hepatic fibrosis.” All duplicate entries were eliminated, resulting in a non-redundant list of disease-associated targets. The herbs-active components-targets network was constructed and visualized via the Cytoscape 3.7.1 software after the names were standardized using the Uniprot database (https://www.uniprot.org, release 2024-05).

#### 2.7.2 Coincident targets identification and protein-protein interaction network analysis

Cross-sectional analysis was conducted to identify the overlapping targets between bioactive components of PZH and fibrosis-related targets using the Venny 2.1.0 software (https://bioinfogp.cnb.csic.es/tools/venny/index.html). These common targets were considered as potential therapeutic targets for PZH in treating liver fibrosis. The search tool for the retrieval of interacting genes(STRING) database (https://string-db.org, version 12.0) (22) was used to construct protein–protein interaction (PPI) networks with a medium confidence score threshold and species restriction to Homo sapiens. The resulting data was then imported into Cytoscape 3.7.2 for network visualization.

#### 2.7.3 Gene ontology function and Kyoto encyclopedia of genes and genomes enrichment analysis

The BioMart (https://www.ensembl.org/biomart/martview) software was used to convert the common targets to Gene Stable IDs. Gene Ontology (GO) enrichment analysis (biological processes, molecular functions, and cellular components) and KEGG pathway analyses were performed using the OmicShare platform (https://www.omicshare.com). Significantly enriched terms (*P* < 0.05) were visualized as bar plots and bubble charts to highlight key pathways and biological functions associated with the anti-fibrotic effects of PZH.

#### 2.7.4 Molecular docking for ginsenoside Rh2

Molecular docking simulations were conducted to elucidate the binding modes and affinities of ginsenoside Rh2 with the core target proteins identified in this study. In specific operations, we first prepared the receptor proteins: EGFR, JAK1, and STAT3 were selected as target proteins, and high-resolution crystal structures of Homo sapiens origin were retrieved from the Protein Data Bank (PDB) (EGFR (PDB ID: *1M14*), JAK1 (PDB ID: *6SM8*), and STAT3 (PDB ID: *6NJS*)). After docking, we systematically analyzed the binding energy scores and interaction patterns to identify the optimal conformation. Prior to docking, the protein structures underwent preprocessing in PyMOL (23) to remove crystallographic water molecules and any co-crystallized ligands. Subsequently, the prepared proteins were imported into AutoDock Tools 1.5.7 for protonation at pH 7.4, assignment of atomic charges (24), and merging of non-polar hydrogen atoms. Subsequently, the grid dimensions for docking of compound ginsenoside Rh2 with STAT3, JAK1, and EGFR were set as 71.25 × 108.75 × 102.75 Å, 112.5 × 97.5 × 63.75 Å, and 102.0 × 75.0 × 47.25 Å, respectively—covering the central region of the protein active site. Parameter settings included output of the top 20 conformations (n_modes = 20), an energy cutoff threshold of 4 kcal/mol, and an exhaustiveness value of 10. Blind docking calculations were performed using Vina to comprehensively scan the protein surface. After docking, we systematically analyzed the binding energy scores and interaction patterns to identify the optimal conformation.

#### 2.7.5 Molecular dynamics simulation

Based on the molecular docking results, classical molecular dynamics (MD) simulations were performed using GROMACS (version 2023.5)(https://www.gromacs.org/) (25) to validate the binding interactions between ginsenoside Rh2 and EGFR, JAK1, and STAT3. The topology of each protein was generated using the pdb2gmx module with the CHARMM36 (26) force field. Each protein was solvated in a dodecahedral box with a TIP3P water (27)model, maintaining a minimum distance of 1.0 nm between the protein and the box boundaries in all directions. Sodium (Na^+^) and chloride (Cl^−^) ions were added to neutralize the system and mimic physiological ionic conditions. Energy minimization was conducted using the steepest descent algorithm with a Verlet cut-off scheme for up to 50,000 steps to achieve a stable energy-minimized configuration. The system was subsequently equilibrated under canonical (NVT) and isothermal-isobaric (NPT) ensembles for 100 ps each. Temperature was maintained at 300 K using the V-rescale thermostat, an improved variant of the Berendsen thermostat, with a coupling constant of 0.1 ps (28). Pressure was regulated at 1 bar using the Parrinello-Rahman barostat, employing a coupling constant of 2.0 ps and a compressibility of 4.5 × 10^−5^ bar^−1^ (29). Alternatively, the C-rescale pressure coupling algorithm was also applied under the same conditions to maintain constant pressure (30). Long-range electrostatic interactions were treated using the Particle Mesh Ewald (PME) method, while short-range Coulomb and van der Waals interactions were computed with a cut-off of 1.2 nm. Bond constraints were applied using the LINCS algorithm (31). Production MD simulations were carried out for 100 ns per complex. Trajectory analysis was performed using built-in GROMACS utilities. For all root mean square deviation (RMSD) analyses, the protein backbone atoms including C, Cα, N of each simulated complex were first least-squares fitted to the backbone atoms of the initial energy-minimized structure to remove global translational and rotational effects. The RMSD was then calculated for the same set of fitted backbone atoms to quantify the conformational stability of the protein throughout the simulation trajectory. In addition, the ligand RMSD for ginsenoside Rh2 was computed after fitting the trajectory onto the protein backbone to assess the stability of the ligand within the binding pocket. Key structural and dynamic properties were evaluated, including the root mean square deviation (RMSD) (32), root mean square fluctuation (RMSF) (33), radius of gyration (Rg) (34), solvent-accessible surface area (SASA) (35), and intermolecular hydrogen bonds between the ligand and protein. To quantitatively assess binding energetics, the gmx MMPBSA tool was employed. This method estimates the binding free energy by combining molecular mechanics (MM) energy terms including van der Waals and electrostatic contributions with implicit solvation effects, decomposed into polar and non-polar components. The polar solvation energy was calculated using the Poisson–Boltzmann equation, while the non-polar contribution was estimated based on the SASA. The overall gas-phase MM energy, total solvation energy, and restraint energy were also computed. The MMPBSA analysis was performed over 2000 frames extracted from the last 20 ns of each stable trajectory (36), using an integrated PBSA solver. Results were interpreted and visualized using the MMPBSA_ana module. All MD and MMPBSA calculations were executed via automated workflows implemented on the ScientiFlow platform (https://scientiflow.com/), ensuring reproducibility and computational efficiency.

### 2.8 Cell culture and treatment

RAW264.7 cells (ATCC^®^ TIB-71™) were obtained from Wuhan Punosai Life Sciences Co Ltd. (Wuhan, China) and authenticated via short tandem repeat (STR) profiling. The cells were cultured in Dulbecco’s Modified Eagle Medium (DMEM) with 10% fetal bovine serum (FBS), and then incubated in a 5% CO_2_ humidified atmosphere at 37°C. After inoculated in 96-well or 6-well culture plates, cells were divide into groups as: vehicle control (VC, 1‰ DMSO solution), model (LPS), PZH high-dose (100 μg/mL), PZH medium-dose (75 μg/mL), PZH low-dose (50 μg/mL), ginsenoside Rh2 high-dose (6 μg/mL), ginsenoside Rh2 medium-dose (4 μg/mL), and ginsenoside Rh2 low-dose (2 μg/mL). 2 h after pre-administration in the administration group, both the model group and the administration group were treated with 1 μg/mL LPS for 24 h.

### 2.9 Immunofluorescence staining for IRF5 expression in M1 macrophages

Cells were cultured in the 24-well plate at 1 × 10^4^ cells/well for 24 h. After 2 h of pre-administration in the treatment groups, the model group and treatment groups were incubated for 1 μg/mL LPS for 24 h. They were then fixed, blocked, and incubated with the IRF5 antibody at 4°C overnight, and then treated with a fluorescent secondary antibody for 1 h at 37°C. The cell nuclei were stained with DAPI for 5~10 min. Finally, the images were captured using a fluorescent inverted microscope.

### 2.10 Enzyme-linked immunosorbent assay for TNF-α, IL-6, and IL-1β

Cells were cultured in 24-well plates at 1 × 10^4^ cells/well. After 2 h of pre-administration in the treatment groups, the model group and treatment groups were incubated for 1 μg/mL LPS for 24 h. supernatants were collected through centrifugation at 1500 g for 5 min. The concentrations of TNF-α, IL-6, and IL-1β were measured using ELISA kits following the manufacturer’s instructions.

Additionally, TNF-α, IL-6, and IL-1β were also determined *in vivo*. Blood samples were collected from the mice and then centrifuged at 1500 × g for 10 min to obtain serum. The levels of TNF-α, IL-6, and IL-1β in the serum were measured using ELISA kits strictly in accordance with the manufacturer’s instructions.

### 2.11 Griess assay for nitric oxide quantification in RAW 264.7 cell supernatants

Cells were cultured in 24-well plates at 1 × 10^4^ cells/well. After 2 h of pre-administration in the treatment groups, the model group and treatment groups were incubated for 1 μg/mL LPS for 24 h. Next, 50 μL of culture supernatant was transferred to 96-well plates. Equal volumes (50 μL) of Griess reagent A and Griess reagent B were sequentially added to each well, followed by further incubation at room temperature in the dark for 10 min to allow chromogenic reaction. The absorbance of the cell supernatant was measured at 540 nm using a microplate reader. Nitrite concentration was calculated using a standard curve generated with sodium nitrite solutions (0~100 μM).

### 2.12 Western blot analysis

Total and nuclear proteins from cells and liver tissues were prepared using standard procedures. The concentration of extracted protein was measured using the bicinchoninic acid (BCA) method. Equal amounts of protein from each group were separated by 10~12% sodium dodecyl sulfate-polyacrylamide gel electrophoresis (SDS-PAGE) and transferred to a polyvinylidene difluoride (PVDF) membrane. The PVDF membrane was blocked with 5% skim milk and incubated with specific primary antibodies targeting the EGFR, JAK1, STAT3, p-JAK1, p-STAT3, CD68, IRF5, IL-12A, and GAPDH proteins overnight at 4 °C. The membrane was washed with TBST and incubated with a horseradish peroxidase-conjugated secondary antibody for 1 h at room temperature. Finally, the membranes were incubated with chromogenic reagents A and B and shaken gently at room temperature for 1 min. PVDF membranes were developed using a chemiluminescence gel imaging system and analyzed using Image Lab software.

### 2.13 Statistical analysis

All statistical analyses were performed using the SPSS 26.0 software (IBM Corp., Armonk, NY, USA). Normality tests were conducted on all the data, with normally distributed data presented as mean ± standard deviation (x^-^ ± s). Differences among the groups were assessed using the one-way analysis of variance (ANOVA). Prior to ANOVA, the homogeneity of variances was evaluated using Levene’s test. For data with homogeneous variance (Levene’s test,*P*>0.05), the least significant difference (LSD) *post-hoc* test was conducted. For data that violated the assumption of homogeneity of variance (Levene’s test, *P*≤ 0.05), the Games-Howell *post-hoc* test was applied. Non-normally distributed data were analyzed using the Kruskal-Wallis H test, which is a non-parametric multiple-sample rank sum test. Statistical significance was set at p<0.05. GraphPad Prism (9.5) was used for data visualization.

## 3 Results

### 3.1 PZH attenuated body weight and liver fibrosis markers

**Figure 1:**
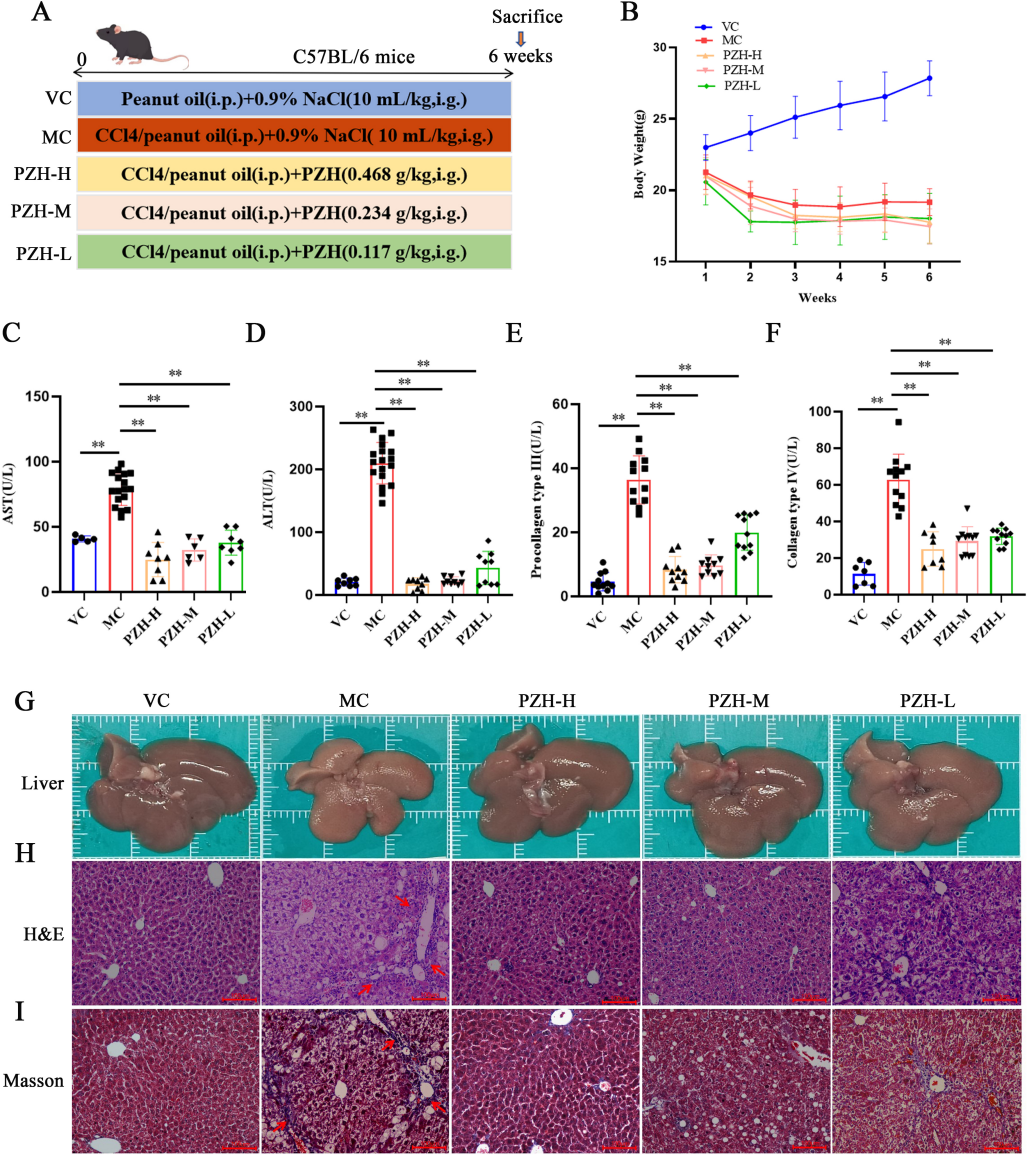
The Effects of PZH in CCL4-Induced Mice: Body Weight, Liver Fibrosis Markers, and Histological Changes. **(A)** Animal experiment procedures. **(B)** Body weight changes during the whole experiment. **(C)** Serum AST. **(D)** Serum ALT. **(E)** Serum C-IV. **(F)** Serum PC-III. **(G)** Representative images showing the morphology. **(H)** H&E staining. **(I)** Masson’strichrome staining. Red arrows indicates the damaged liver tissue and fiber cords. (H&E 200 ×; Masson 200 ×). Data are presented as the mean ± SD with significance markers of ^**^ *P* < 0.01.

All the experimental procedures are shown in **Figure 1A**. The VC mice exhibited a steady gain in body weight (**Figure 1B**). In contrast, all groups receiving CCL_4_ injections showed progressive weight loss following the initial administration, indicating systemic toxicity associated with hepatic fibrosis induction. Notably, there were no significant changes in the weight loss following treatment with PZH compared to the model group. After 6 weeks of CCL_4_ administration, serum levels of AST, ALT, procollagen type III (PC-III), and collagen type IV (C-IV) in the MC mice were significantly increased compared to the VC group (*P* < 0.01). Conversely, PZH treatment significantly reduced the levels of serum AST, ALT, PC-III, and C-IV in a dose-dependent manner compared to the MC group (*P* < 0.01) (**Figures 1C–F**). This significant attenuation of both hepatocellular injury markers and extracellular matrix components demonstrates the multifaceted anti-fibrotic efficacy of PZH.

### 3.2 PZH improved hepatic histopathology and attenuated collagen deposition in fibrotic mice

Gross and histological examinations demonstrated that PZH has protective effects against CCL_4_-induced hepatic damage. Macroscopically, the livers of the VC group were healthy and reddish with smooth surfaces, whereas the liver of the MC group displayed characteristic fibrotic changes, including yellowish-brown discoloration, rough granular surfaces, and a hardened texture. Notably, treatment with PZH in a dose-dependent manner improved liver morphology, with high- and medium-dose groups showing near-normal coloration and smooth surfaces (**Figure 1G**). These findings were validated through histological evaluation using H&E staining: the normal hepatic lobular architecture with radially arranged hepatocyte cords was preserved in the VC group, while the MC group exhibited severe hepatocyte ballooning, inflammatory infiltration, and fibrotic septa formation. These pathological changes were significantly ameliorated through PZH administration, with high- and medium-dose groups showing significantly reduced cellular edema and inflammatory cell infiltration (**Figure 1H**). These observations were validated through Masson’s trichrome staining, demonstrating extensive collagen deposition (blue staining) and architectural distortion in the MC mice livers compared to minimal staining in the VC group. Notably, PZH treatment dose-dependently reduced collagen accumulation, with high- and medium-dose groups exhibiting near-normal collagen levels, with preserved lobular morphology (**Figure 1I**). Collectively, these findings demonstrate that treatment with PZH improves liver function and attenuates inflammatory pathological lesions.

### 3.3 PZH modulated hepatic macrophage polarization and suppressed pro-inflammatory activation in liver fibrosis

**Figure 2:**
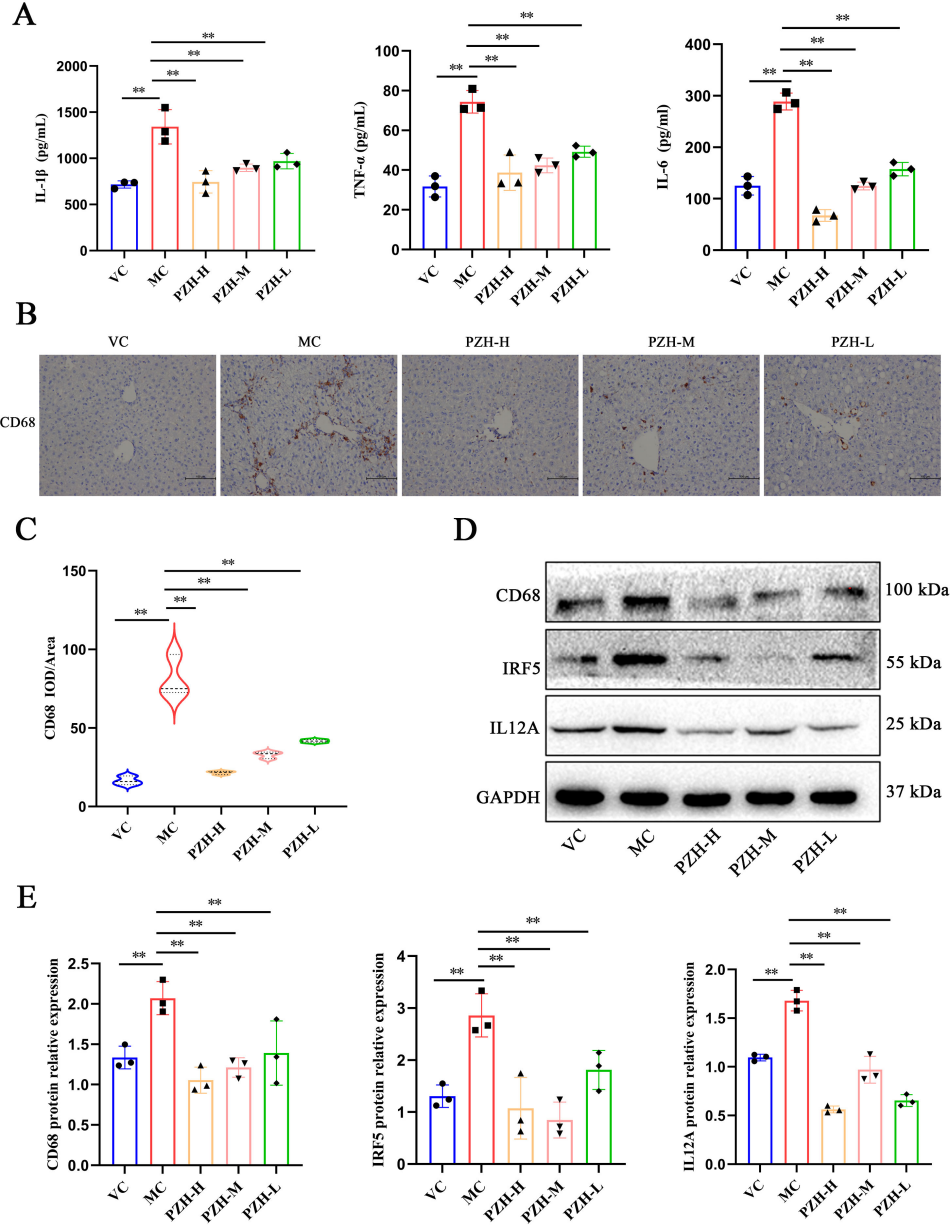
The impact of PZH on pro-inflammatory cytokine production and M1 macrophage polarization in the liver of fibrotic mice. **(A)** The expression levels of IL-1β, IL-6, and TNF-α in mice serum quantified by ELISA assays. **(B)** Immunohistochemistry staining of CD68. **(C)** Expression levels of CD68 in liver quantified by Immunohistochemistry assays. **(D)** Western blot analysis of CD68, IRF5, IL12A protein. **(E)** Quantitation of western blot analysis of CD68, IRF5, and IL12A proteins. Data are presented as the mean ± SD with significance markers of ^**^ *P* < 0.01.

To gain a deeper understanding of the inflammatory microenvironment associated with fibrosis progression, we conducted a quantitative analysis of key pro-inflammatory cytokines. The results obtained from the ELISA assay indicated that the levels of TNF-α, IL-6, and IL-1β were significantly higher in the MC mice relative to the VC group (*P* < 0.01). Notably, administration of PZH resulted in a marked, dose-dependent reduction in the production of these cytokines (*P* < 0.01), thereby demonstrating a potent anti-inflammatory effect (**Figure 2A**). Immunohistochemical analysis revealed significant alterations in the population of the macrophages during fibrogenesis. Notably, MC mice exhibited markedly increased CD68 macrophage infiltration compared to the VC group (*P* < 0.01), indicative of enhanced monocyte recruitment during hepatic fibrosis. The expression of CD68 were significantly reduced in a dose-dependent manner using PZH treatment (*P* < 0.01), indicative of inhibited macrophage hepatic infiltration (**Figures 2B, C**). Furthermore, PZH significantly attenuated pro-inflammatory macrophage polarization, as evidenced by decreased expression of IRF5 (*P* < 0.01) and IL-12A (*P* < 0.01) compared to the MC mice (**Figures 2D, E**). The coordinated downregulation of pro-inflammatory markers—CD68, IRF5, and IL-12A, indicates that PZH suppressed M1-like state.

### 3.4 Network pharmacology predicts that PZH may affect liver fibrosis in HSCs via the EGFR/JAK1/STAT3 signaling pathway

**Figure 3:**
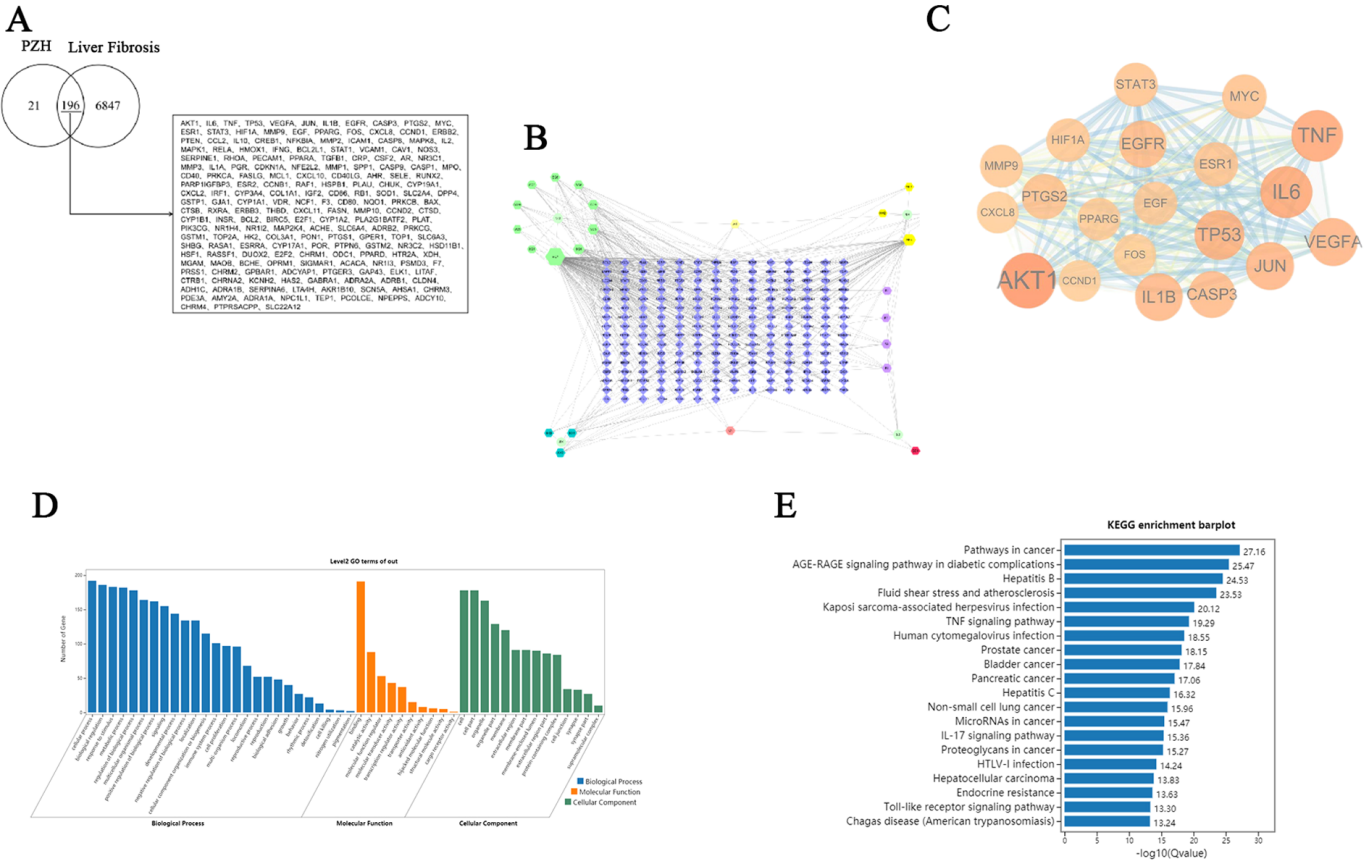
Network pharmacology analysis for the PZH prevention and treatment of liver fibrosis. **(A)** Venny diagram of overlapping genes from PZH and liver fibrosis. **(B)** Drug-target-disease network. **(C)** PPI network construction. the darker the color, the larger the circle, and the more significant it is. **(D)** GO functional enrichment analysis. **(E)** KEGG pathway enrichment analysis.

Through systematic screening of the TCMSP database and literature mining, we identified 31 bioactive compounds in PZH. After the elimination of duplicate entries, a total of 24 unique components were identified. The pharmacologically active constituents of the formulation were derived from its four principal herbal components: *Panax notoginseng* (11 compounds), *Calculus bovis* (10 compounds), *Moschus* (4 compounds), and *Serpentis Bill* (6 compounds) (**Table 1**). Key bioactive constituents were prioritized based on network centrality parameters (degree centrality and betweenness centrality), with quercetin, ursolic acid, β-sitosterol, stigmasterol, β-elemene, lauric acid, ginsenoside Rh2, testosterone, 17-β-estradiol, and ursodeoxycholic acid as the most pharmacologically relevant components. Online databases were used to identify the potential targets of liver fibrosis and PZH. A total of 217 target genes were identified for PZH, while 7043 target genes were identified for liver fibrosis. Among these, there were 196 common targets, representing 90.32% of the overlap, indicated a high correlation between PZH targets and liver fibrosis targets, suggesting that PZH has therapeutic potential for liver fibrosis (**Figure 3A**). The processed PPI network was visualized using Cytoscape 3.7.1 software (**Figures 3B, C**), with strict adherence to the selection criteria, thereby resulting in a total of 20 core targets, including AKT1, IL6, TNF, TP53, VEGFA, JUN, IL1B, EGFR, CASP3, PTGS2, MYC, ESR1, STAT3, HIF1A, MMP9, EGF, PPARG, FOS, CXCL8, and CCND1, which are considered key targets of contributing to the anti-hepatic fibrosis activity of PZH. To explore the molecular mechanisms of the anti-hepatic fibrosis effect of PZH, GO, and KEGG pathway enrichment analysis were performed on the common targets between PZH and hepatic fibrosis. The GO enrichment analysis results established 6206 biological processes (BPs) related to regulation of metabolic processes, immune system processes, and response to exogenous stimuli (**Figure 3D**). The core targets were linked to 908 molecular functions (MFs), including antioxidant activity, and 537 cellular components (CCs), such as those involved in cell proliferation. KEGG pathway analysis revealed 175 signaling pathways associated with PZH and liver fibrosis were obtained, encompassing pathways associated with cancer, glucose metabolism, hepatitis B, and cytokine signaling pathways (**Figure 3E**). Of which, core targets (EGFR, STAT3, JAK1) all are enriched in cancer signaling pathways, given their high connectivity in the PPI network (**Figure 3C**) and established crosstalk in liver fibrosis, we hypothesized that PZH modulates the EGFR/JAK1/STAT3 signaling axis. Subsequently, we selected this pathway for further analysis in both *in vitro* and *in vivo* experiments.

**Table 1:** Main chemical components of PZH.

Traditional Chinese Medicines	Components
Panax notoginseng	D-Mannitol; Liquiritigenin; Quercetin; Oleanolic acid; β-Sitosterol; Stigmasterol; Ginsenoside Rh2; Lauric acid; Diisooctyl phthalate; β-Elemene; α-Cedrene
Calculus Bovis	Methyl deoxycholate; Ursolic acid; Oleanolic acid; Deoxycholic acid; Taurocholic acid; Chenodeoxycholic acid; Ursodeoxycholic acid; Cholesterol; (8S,9S,10R,13S,14R,17S)-17-(2-hydroxyacetyl)-10,13-dimethyl-1,2,6,7,8,9,11,12,14,15,16,17-dodecahydrocyclopenta[a]phenanthren-3-one; methyl(4R)-4-[(3R,5S,7S,8R,9S,10S,12S,13R,14S,17R)-3,7,12-trihydroxy-10,13-dimethyl-2,3,4,5,6,7,8,9,11,12,14,15,16,17-tetradecahydro-1H-cyclopenta[a]phenanthren-17-yl]pentanoate
Moschus	17β-Estradiol; Testosterone; Moronic acid; Cholesterol
Serpentis Fel	Taurochenodeoxycholic acid; Deoxycholic acid; Taurocholic acid; Chenodeoxycholic acid; Ursodeoxycholic acid

### 3.5 Molecular docking results

**Figure 4:**
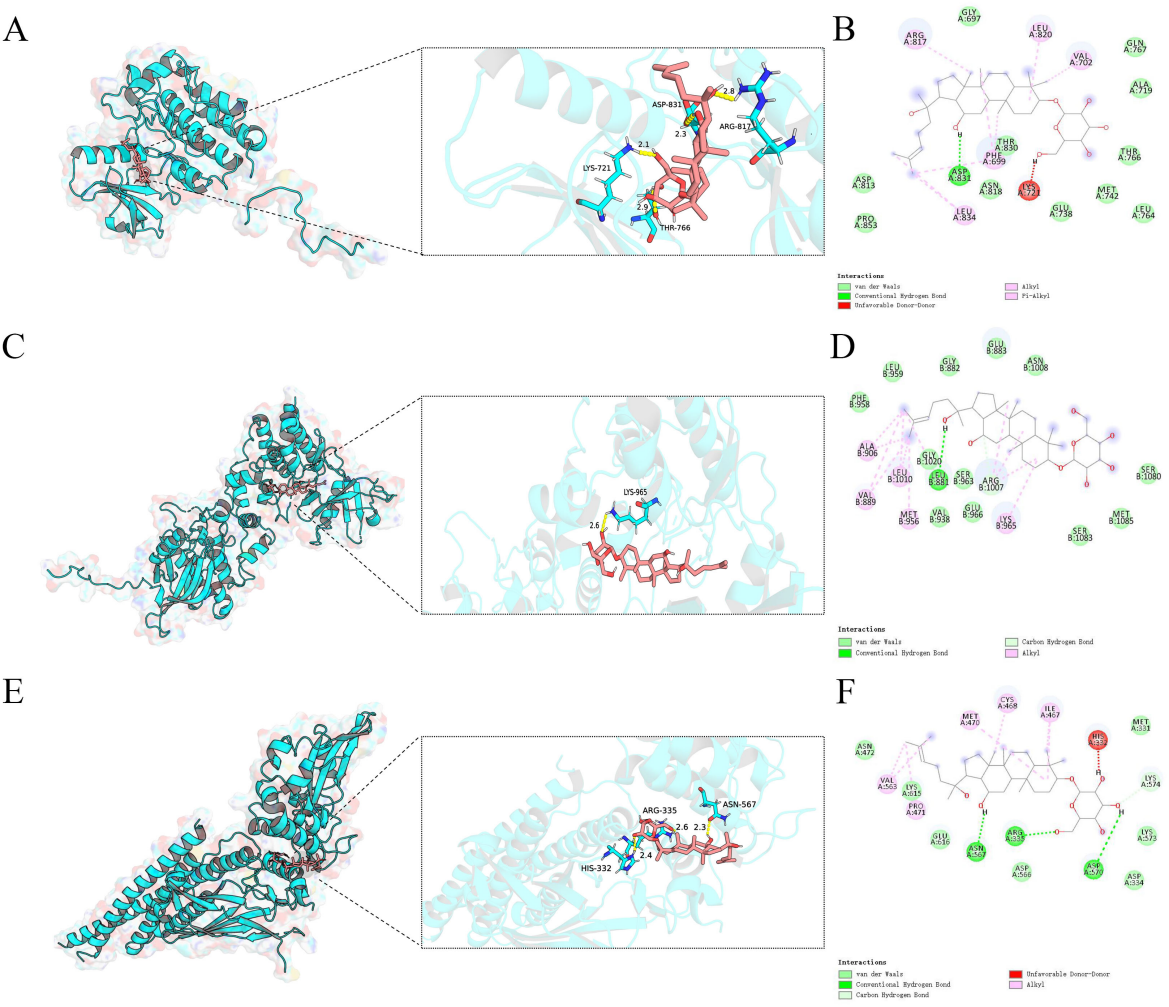
Molecular docking analysis of ginsenoside Rh2 bound to EGFR,JAK1,STAT3. Ginsenoside Rh2 bound to EGFR,JAK1,STAT3 shown as 3D diagrams. **(A)** Ginsenoside Rh2-EGFR; **(C)** Ginsenoside Rh2-JAK1; **(E)** Ginsenoside Rh2-STAT3; **(B, D, F)** ginsenoside Rh2 bound to EGFR, JAK1, STAT3 shown as 2D diagrams.

A molecular docking analysis was performed to examine the interactions between ginsenoside Rh2 and three core target genes: EGFR, JAK1, and STAT3. Utilizing AutoDock software, docking simulations were conducted, revealing low binding energies that suggest strong affinities between the compound and the target proteins. The docking fraction of the compound and the protein is shown in **Figure 4** depicts an image of the optimal docking of receptor and ligand after visualization. The results showed that ginsenoside Rh2 could interact with EGFR, JAK1 and STAT3. (**Figures 4A–F**). **Figures 4A, B** shows that ginsenoside Rh2 forms one hydrogen bond with ASP831, ARG817, LYS721, and THR766 in EGFR. **Figures 4C, D** shows that ginsenoside Rh2 forms one hydrogen bond with LYS965 in JAK1. **Figures 4E, F** shows that ginsenoside Rh2 can interact with HIS332, ARG335, ASN567 in STAT3 through three hydrogen bond. Notably, all three core target proteins demonstrated significant binding to ginsenoside Rh2, with binding energies all below zero. Specifically, the calculated binding energies for ginsenoside Rh2 with EGFR, JAK1, and STAT3 were -8.042, -9.444, and -8.085 kcal/mol, respectively. These values indicate spontaneous binding, underscoring the potential roles of these interactions in the molecular mechanisms underlying liver injury.

### 3.6 Molecular stability of docked complexes

**Figure 5:**
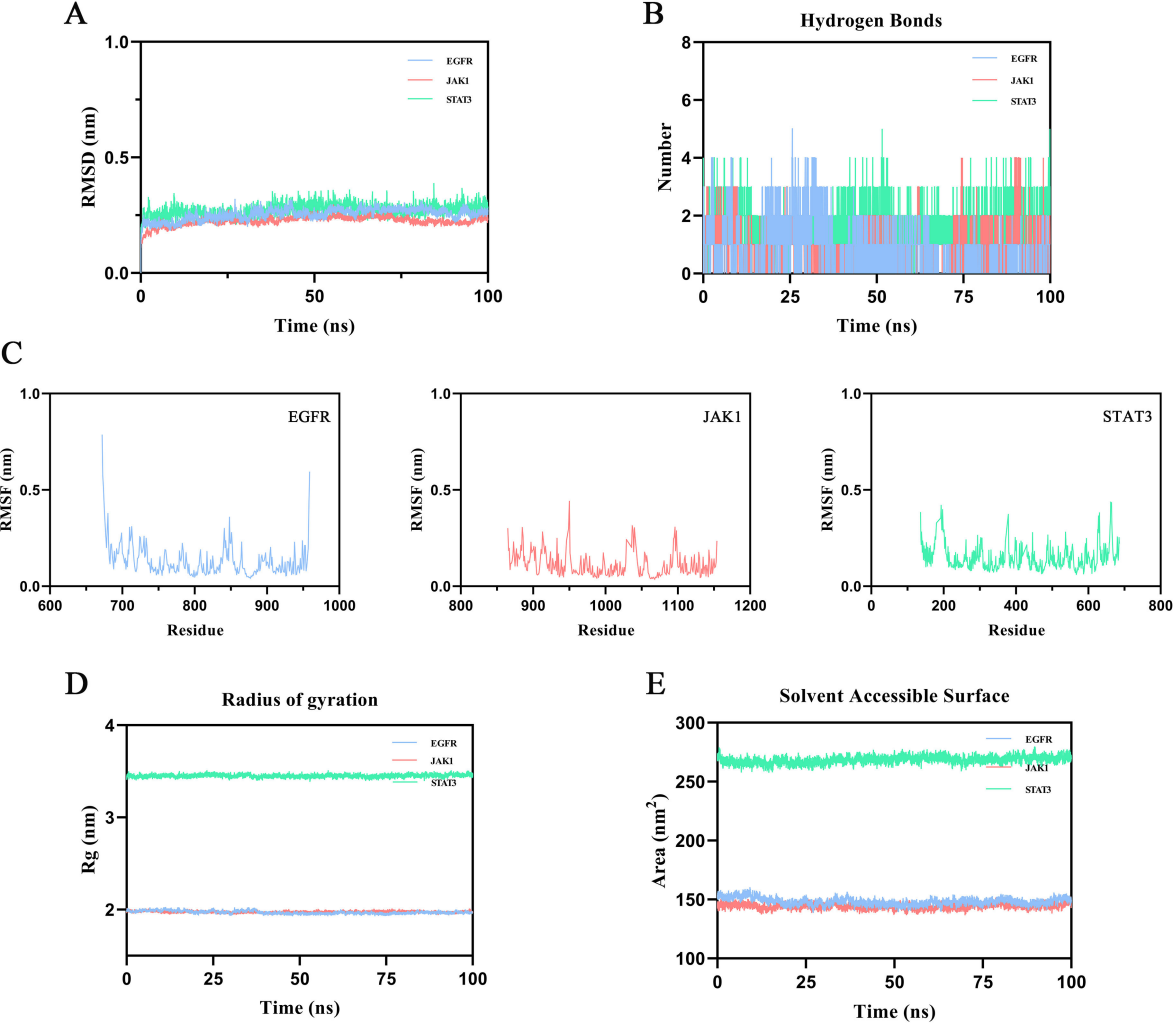
Results of molecular dynamics (MD) simulation. **(A)** Root mean square deviation (RMSD). Intensely fluctuated residues are highlighted **(B)** Number of hydrogen bonds across the simulation period. **(C)** Root mean square fluctuation (RMSF). **(D)** Radius of Gyration (Rg). **(E)** Solvent-accessible surface area (SASA).

**Figure 6:**
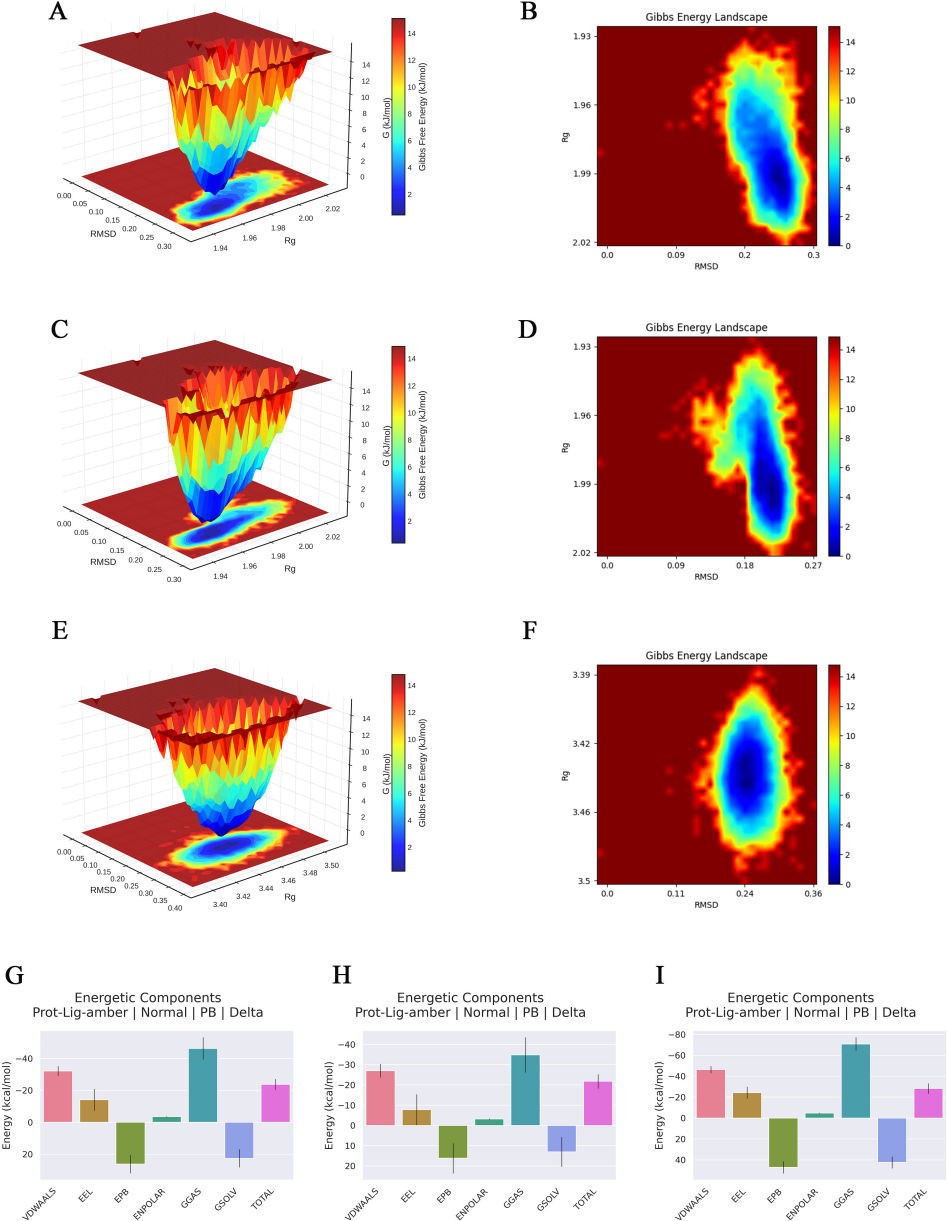
Gibbs free energy landscape and MMpbsa based energy component analysis of three types of complexes. **(A, C, E)** Three-dimensional Gibbs free energy landscapes of the ginsenoside Rh2-EGFR, Rh2-JAK1, and Rh2-STAT3. **(B, D, F)** Two-dimensional Gibbs free energy landscapes of the ginsenoside Rh2-EGFR, Rh2-JAK1, and Rh2-STAT3. A color gradient from red standing for high free energy to blue standing for low free energy was used to represent energy values. Blue regions correspond to energy minima, indicative of the most stable and compact conformational states. **(G–I)** Decomposition of the MM/PBSA binding free energy for the complexes of ginsenoside Rh2 with EGFR, STAT3, and JAK1.

To evaluate the binding stability of ginsenoside Rh2 with EGFR, JAK1, and STAT3, MD simulations were performed for 100 ns on each complex. Structural stability was assessed using multiple quantitative metrics, including RMSD, hydrogen bonding, RMSF, Rg, and SASA. The RMSD of the protein backbone atoms was calculated to monitor conformational stability over time. As depicted in **Figure 5A**, each system reached equilibrium within the final 20 ns of the simulation. The RMSD values fluctuated within a minimal range of 0.2 nm, indicating that Rh2-EGFR, Rh2-JAK1, and Rh2-STAT3, achieved structural equilibrium and remained stable throughout the simulation period. Hydrogen bonding analysis is important in featuring ligand-protein binding, as hydrogen bonds can steer the binding strength of small molecules. Through the built-in module of GROMACS software, we calculated the number and the population of hydrogen bonds between ligands and proteins in a time-dependent manner (**Figure 5B**). The average numbers of hydrogen bonds between Rh2 and EGFR, JAK1, and STAT3 were 0.756, 0.928, and 1.526, respectively, with maximum intermolecular hydrogen bonds reaching 5, 4, and 5 during the simulation. These results suggest the presence of a stable hydrogen-bonding network that enhances binding affinity and specificity. To evaluate local flexibility, per-residue RMSF was analyzed. As illustrated in **Figure 5C**, most residues in all three complexes exhibited low fluctuations (< 0.3 nm), suggesting reduced flexibility and increased rigidity upon Rh2 binding. This attenuation of residual mobility further supports the stabilizing effect conferred by ligand interaction. The Rg was employed to assess the global compactness and structural integrity of the proteins. Throughout the simulation, the Rg values for Rh2-EGFR, Rh2-JAK1, and Rh2-STAT3 complexes remained confined within narrow ranges of 1.933~2.027 nm, 1.945~2.014 nm, and 3.392~3.511 nm, respectively (**Figure 5D**). These consistently low profiles suggest no significant unfolding or structural loosening occurred, indicating well-maintained compactness in all complexes. Furthermore, the SASA was monitored to probe changes in surface exposure and hydrophobic core packing. The SASA values fluctuated mildly within the ranges of 139.04~159.90 nm², 136.59~152.63 nm², and 258.03~279.50 nm² for the three complexes (**Figure 5E**), implying that the hydrophobic cores remained buried and no major structural rearrangements took place. To gain deeper insights into the energetic stability and conformational ensembles of the complexes, the free energy landscape (FEL) was constructed using RMSD and Rg as reaction coordinates. RMS captured conformational departure from the initial structure, while Rg reflected overall compactness. The FEL was built based on three-dimensional data involving RMSD, Rg, and Gibbs free energy, and visualized both as 3D surfaces and 2 contour plots (**Figures 6A–F**). A color gradient from red standing for high free energy to blue standing for low free energy was used to represent energy values. Blue regions correspond to energy minima, indicative of the most stable and compact conformational states. The Gibbs free energy values for the stabilized ensembles of Rh2–EGFR (**Figures 6A, B**), Rh2–JAK1 (**Figures 6C, D**), and Rh2–STAT3 (**Figures 6E, F**) complexes were found to lie between 0 and 3.35 kcal/mol. Each system exhibited at least one pronounced global energy minimum, signifying a thermodynamically favorable state. These results confirm that all complexes achieved energetically stable conformations, further supporting the structural stability inferred from the MD simulations from a thermodynamic perspective.

### 3.7 Molecular mechanics Poisson-Boltzmann surface area analysis

The MM/PBSA binding free energy calculations were performed using the last 10 ns of the stabilized trajectory from the molecular dynamics simulation of each complex. One frame per 10 frames was extracted, yielding a total of 150 frames for the computation. The results are presented in **Figures 6G–I**, the binding free energies and their components for the complexes of ginsenoside Rh2 with EGFR, JAK1, and STAT3 are summarized in **Table 2**. The total binding free energies (ΔTOTAL) were calculated to be –21.78 kcal/mol, –23.74 kcal/mol, and –28.30 kcal/mol for the EGFR, JAK1, and STAT3 complexes, respectively, indicating strong spontaneous binding in all cases. The van der Waals interactions (ΔVDWAALS) and electrostatic contributions (ΔEEL) were major favorable drivers for complex formation, with values of –27.03 and –7.76 for EGFR, –32.16 and –14.14 for JAK1, and –46.38 and –24.37 for STAT3. The gas-phase free energy (ΔGGAS) was also highly favorable across all systems. Conversely, the polar solvation term (ΔEPB) opposed binding, whereas the nonpolar solvation component (ΔENPOLAR) contributed favorably. These energy decompositions provide thermodynamic insight into the binding mechanisms and affirm the robustness of the interactions between ginsenoside Rh2 and the target proteins.

**Table 2:** Calculated binding free energy via MM/PBSA strategy.

Energy Component	EGFR-complex (Kcal/mol)	JAK1-complex (Kcal/mol)	STAT3-complex (Kcal/mol)
△VDWAALS	-27.03	-32.16	-46.38
△EEL	-7.76	-14.14	-24.37
△EPB	16.20	26.14	47.19
△ENPOLAR	-3.19	-3.58	-4.73
△EDISPER	0.00	0.00	0.00
△GGAS	-34.79	-46.30	-70.76
△GSOLV	13.01	22.55	42.46
△TOTAL	-21.78	-23.74	-28.30

All energy values are in kcal/mol.

### 3.8 PZH and ginsenoside Rh2 suppress LPS-induced inflammatory cytokine secretion in RAW264.7 macrophages

**Figure 7:**
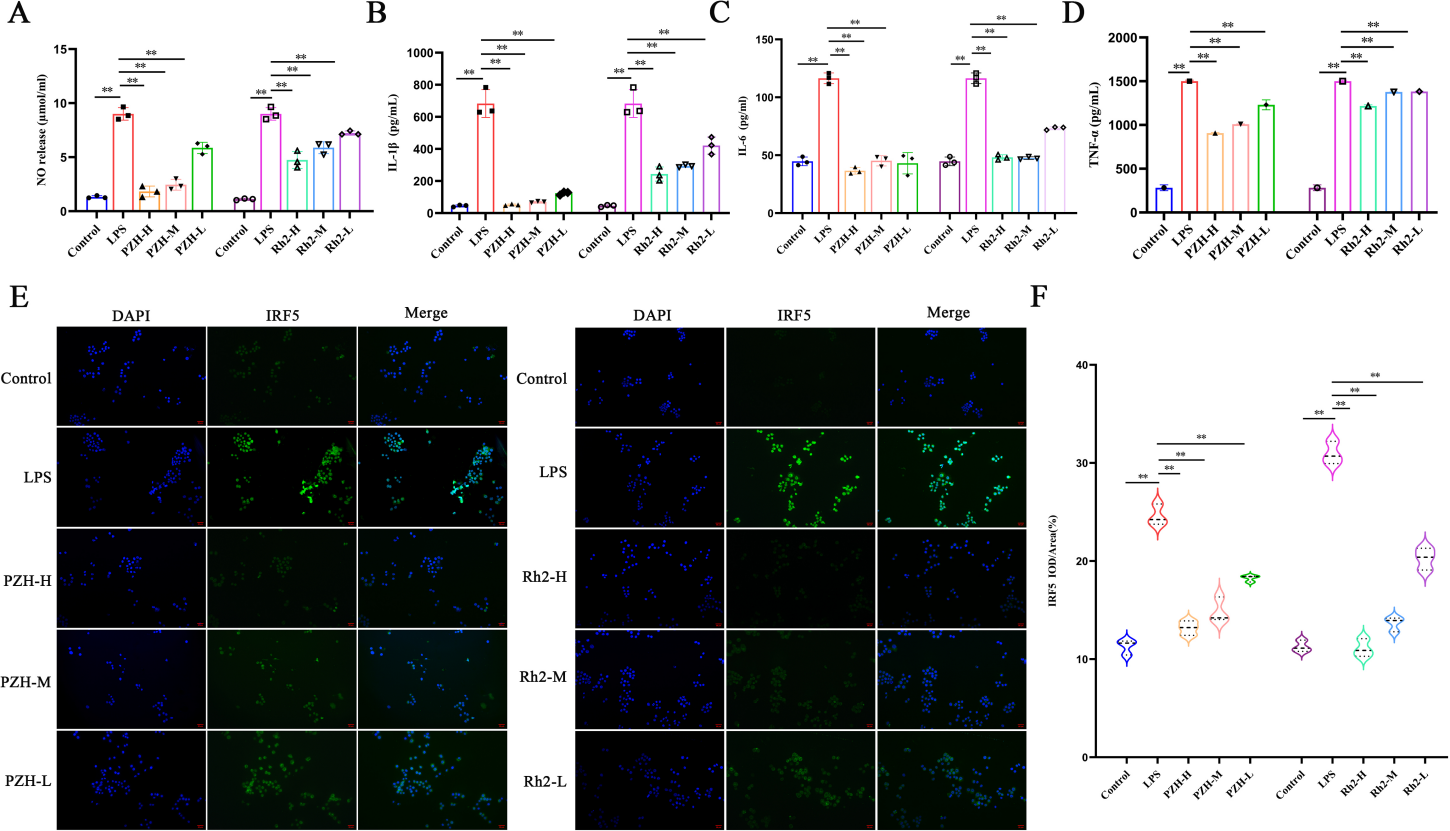
Effects of PZH and Ginsenoside Rh2 on RAW 264.7 macrophages as determined by Immunohistochemistry, Griess and ELISA analysis. **(A)** Expression levels of NO in RAW 264.7 macrophages quantified by Griess assays. **(B–D)** The expression levels of IL-1β, IL-6, and TNF-α in RAW 264.7 macrophages quantified by ELISA assays. **(E)** Immunohistochemistry images at the magnification of 200 ×. **(F)** Expression levels of IRF5 in RAW 264.7 macrophages quantified by Immunohistochemistry assays. The data are presented as the mean ± SD with significance markers of ^**^ *P* < 0.01.

Nitric oxide (NO) production in RAW 264.7 macrophages was significantly increased through LPS stimulation compared to the VC group (*P* < 0.01). However, treatment with PZH and ginsenoside Rh2, under all tested doses, significantly reduced NO levels in a dose-dependent manner (*P* < 0.01) (**Figure 7A**). Additionally, LPS induction triggered robust secretion of pro-inflammatory cytokines TNF-α, IL-6, and IL-1β (*P* < 0.01), whereas both PZH and ginsenoside Rh2 significantly attenuated their release (*P* < 0.01), with efficacy significantly depended on the dose (**Figures 7B–D**).

### 3.9 PZH and ginsenoside Rh2 attenuate LPS-induced M1 polarization in RAW264.7 macrophages via suppression of IRF5 expression

Compared to the VC group, LPS stimulation significantly upregulated the expression of IRF5—a key transcription factor driving M1 polarization—in RAW264.7 macrophages, confirming successful induction of pro-inflammatory M1 polarization. However, at all the tested doses, treatment with PZH and ginsenoside Rh2 significantly suppressed IRF5 expression compared to the LPS-induced model group (*P* < 0.01) (**Figures 7E, F**). Both interventions exhibited dose-dependent and statistically significant inhibitory effects. These findings suggest that PZH and ginsenoside Rh2 effectively attenuate LPS-induced M1 macrophage polarization by modulating IRF5, a key transcriptional regulator of inflammatory macrophage activation.

### 3.10 PZH and ginsenoside Rh2 inhibit the EGFR/JAK1/STAT3 signaling pathway in LPS-stimulated macrophages and hepatic fibrosis models

**Figure 8:**
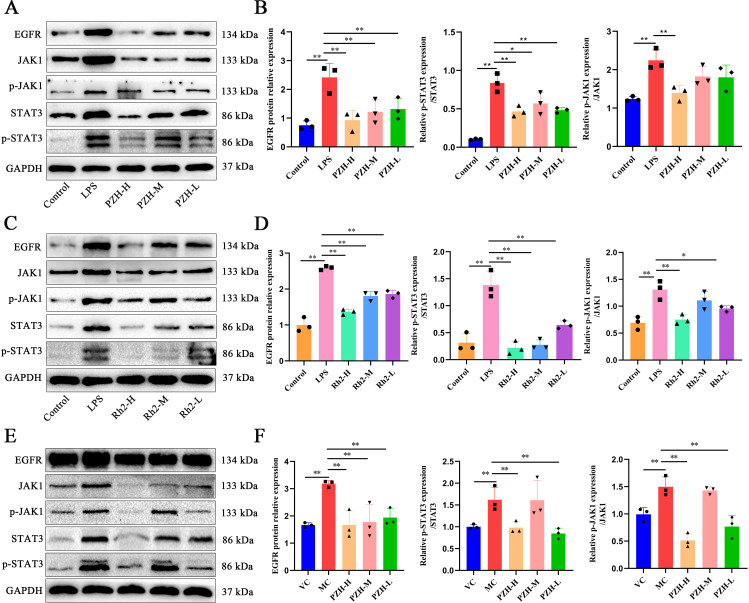
The impact of PZH and Ginsenoside Rh2 on EGFR/JAK1/STAT3 signaling pathway related proteins as determined by Western blot analysis. **(A, C)** In a macrophage model stimulated by LPS, representative Western blot images. **(B, D)** Relative expression levels of EGFR to GAPDH and ratio of p-JAK1/JAK1, p-STAT3/STAT3 in the RAW 264.7 macrophages were quantified by Western blot assays. **(E)** Representative Western blot images for hepatic fibrosis model stimulated by CCL_4_. **(F)** Relative expression levels of EGFR to GAPDH and ratio of p-JAK1/JAK1, p-STAT3/STAT3 in liver was determined by Western blot assays. Data are presented as the mean ± SD with significance markers of ^**^ *P* < 0.01, ^*^ *P* < 0.05.

Both PZH and ginsenoside Rh2 significantly suppressed the overexpression of EGFR, p-JAK1/JAK1, and p-STAT3/STAT3 proteins in the LPS-induced RAW 264.7 macrophages compared to the LPS model group (*P* < 0.01) (**Figures 8A–D**). Additionally, in the hepatic fibrosis mouse model, fibrotic liver tissues exhibited marked upregulation of EGFR, p-JAK1/JAK1, and p-STAT3/STAT3 protein expression (*P* < 0.01), accompanied by increased phosphorylation of JAK1 and STAT3 (*P* < 0.01). PZH treatment effectively reversed these alterations by reducing both the total protein expression and phosphorylation levels of JAK1 and STAT3 (**Figures 8E, F**). These findings highlight that PZH treatment attenuates liver fibrosis and inflammation via the EGFR/JAK1/STAT3 signaling pathway. These observations are consistent with the findings from the bioinformatics analysis.

## 4 Discussion

Hepatic fibrosis is a progressive disease driven by chronic liver injury, characterized by excessive accumulation of extracellular matrix (ECM) and disruption of hepatic architecture. Currently, the pathogenesis of the disease is incompletely understood, and the available therapeutic options have limited efficacy. PZH, a well-established traditional Chinese medicinal formula, comprises natural compounds such as Calculus Bovis, Moschus, Snake Gallbladder, and Radix Notoginseng. It exerts anti-inflammatory, antioxidant, anti-fibrotic, and hepatoprotective effects. Research has demonstrated that it confers therapeutic effects against diverse liver disorders, such as viral hepatitis, cholestasis, and cirrhosis (37). However, its specific molecular mechanisms in the treatment of fibrosis in hepatic fibrosis have not been extensively elucidated.

Long-term clinical practices have indicated that TCM formulae, containing diverse herbs with multiple active ingredients, can regulate complex pathogenic networks in a comprehensive manner. Studies have shown that PZH exemplifies the TCM holistic approach, which adheres to the principles of “ruler, minister, and auxiliaries” and targeting disease mechanisms by “clearing heat, removing toxins, activating blood circulation, and removing blood stasis.” This formula includes Niuhuang, which is the principal component that clears heat, detoxify, and expel phlegm. Musk (the major ingredient) can activate blood circulation and relieve swelling and pain; while Panax notoginseng and snake gall as adjuvant ingredients, can resolve blood stasis and relieve bile congestion. This synergy reflects the TCM approach to treating liver fibrosis, focusing on clearing heat, resolving blood stasis, and dispersing knots (38). Studies have shown that PZH can treat metabolic diseases such as chronic hepatitis and severe fatty liver (39). Although PZH can prevent and treat liver fibrosis, its mechanisms of action have not been fully explored. This study, therefore, investigated the pharmacological effects of PZH on liver fibrosis and its underlying molecular pathways.

The administration of PZH has been shown to ameliorate CCl_4_-induced liver fibrosis in rats (40). Specifically, PZH effectively inhibits the infiltration of inflammatory cells and the production of pro-inflammatory factors induced by CCl_4_, while concurrently suppressing the activation of the NF-κB pathway. In this study, we further demonstrated that PZH attenuated CCL_4_-induced liver injury in mice, AST, ALT levels and histological analyses, including H&E and Masson’s trichrome staining, corroborated that PZH effectively reversed CCL_4_-induced liver injury, inflammatory infiltration, fibrous tissue proliferation, and collagen deposition. To further confirm the anti-hepatic fibrosis effect of PZH, we selected RAW264.7 cells with similar phenotypes, which were widely used in inflammatory cell model studies, *in vitro* experiments. The hepatic inflammatory response is a hallmark of liver fibrosis. The progression of liver fibrosis is often accompanied by chronic inflammation and macrophage activation, all of which exacerbate fibrosis. Activated macrophages accumulate at the site of liver injury, where they secrete inflammatory cytokines and chemokines, thereby perpetuating the inflammatory response and fibrosis (41). In our study, PZH effectively inhibited the production of NO, TNF-α, IL-6, and IL-1β in LPS-stimulated RAW264.7 macrophages and *in vivo*, which implies that this treatment may exert strong anti-inflammatory effects. The observed downregulation of these cytokines aligns with the noted reduction in macrophage infiltration and polarization, thereby reinforcing the hypothesis that PZH modulates the immune microenvironment to mitigate fibrosis progression.

Cyberpharmacology has been recognized to identify disease-related pharmacotherapeutic targets by synthesizing large datasets for virtual screening based on TCM components and associated symptoms (42). To dissect the potential mechanism by which PZH exerts anti-liver fibrosis, we performed a network pharmacology analysis. We identified 24 active ingredients, including quercetin, ursolic acid, β-sitosterol, stigmasterol, β-elemene, lauric acid, and ginsenoside Rh2. In the subsequent analysis, 196 hub genes associated with liver fibrosis were identified, including AKT1, IL6, TNF, TP53, VEGFA, JUN, IL1B, EGFR, CASP3, PTGS2, MYC, ESR1, STAT3, HIF1A, MMP9, and EGF. These genes participate in diverse metabolic pathways that modulate liver fibrosis and were the cardinal genes in the PPI network, highlighting their importance in the progression of liver fibrosis. EGFR, JAK1, and STAT3 were prioritized among 20 core targets based on functional coherence, clinical relevance, and computational support. This axis represents a well-established cascade in hepatic fibrogenesis, with clinical evidence correlating its activation to the severity of fibrosis. Molecular docking studies have confirmed a high binding affinity of ginsenoside Rh2 to these targets. These predictions were further substantiated by MD simulations, which demonstrated that ginsenoside Rh2 forms stable complexes with all three target proteins. Throughout the 100 ns simulation trajectories, the RMSD of the ligand–protein complexes remained consistently low, and the binding free energy (ΔG) values were significantly negative. These results provide robust structural and energetic evidence supporting the strong binding affinity and stability between ginsenoside Rh2 and the core targets. GO and KEGG pathway enrichment analysis revealed that PZH regulated various biological functions associated with immune response, cell proliferation, and antioxidant activity. The signaling pathways regulated by PZH, included those involved in cancer, glucose metabolism, hepatitis B, TNF, and IL-17. Notably, the core targets such as EGF, EGFR, and STAT3 were enriched in the EGFR/JAK1/STAT3 signaling pathway, which has been linked to the development of liver injury, inflammation, and fibrosis. As with Gefitinib-induced EGFR degradation (43), JAK1 destabilization by SOCS1 (44), or STAT3 autotranscription blockade (45), our molecular docking suggests that high-affinity binders in PZH—particularly ginsenoside Rh2—engage analogous regulatory mechanisms. In the early stages of liver injury, EGF and damage-associated molecular patterns (DAMPs) released from hepatocytes activate the EGFR receptor on HSCs, initiating autophosphorylation, thereby activating JAK1 and STAT3 phosphorylation. This activation triggers HSC transformation into myofibroblasts, inhibiting ECM degradation (46).Molecular docking simulations suggest that ginsenoside Rh2—a bioactive constituent of PZH—may attenuate hepatic fibrosis through modulation of the EGFR/JAK1/STAT3 signaling axis. Collectively, our results suggest that PZH exerts pharmacological effects on liver fibrosis by modulating the EGFR/JAK1/STAT3 signaling pathway.

Activation of the EGFR and JAK1/STAT3 signaling pathways has been shown to promote pro-inflammatory polarization in macrophages, thereby accelerating hepatic fibrosis through mechanisms such as inflammation regulation, oxidative stress, and apoptosis. Preclinical studies indicate that pharmacological inhibition of JAK1/STAT3 signaling can suppress macrophage-driven inflammatory responses (47). And in a murine model of hepatic fibrosis induced by CCL_4_, increased EGFR expression and STAT3 activation in hepatic macrophages were positively associated with M1 macrophage infiltration and collagen deposition (48). These findings collectively suggest that targeting the EGFR and JAK1/STAT3 signaling pathway may offer a therapeutic strategy to modulate macrophage inflammatory phenotypes in fibrotic liver disease. In our experiments, PZH administration significantly reduced the expression of EGFR, JAK1, and STAT3 in liver tissues compared to liver fibrosis mice. *In vitro* experiments showed that PZH reduced the LPS-induced expression of EGFR, JAK1, and STAT3 proteins and inhibited pro-inflammatory macrophage polarization in RAW264.7 cells. These results, were consistent with the bioinformatics findings, implying that PZH modulated liver fibrosis by regulating the EGFR/JAK1/STAT3 signaling pathway and inhibiting macrophage M1 polarization.

The anti-inflammatory and immunomodulatory properties of ginsenoside Rh2, a pivotal bioactive component in Pien Tze Huang, are mediated by its capacity to selectively regulate macrophage polarization and the downstream pro-inflammatory signaling cascades. Recent studies further underscore the pleiotropic effects of saponins in modulating critical signaling cascades. Notably, Saikosaponin-a (SSA), a triterpenoid saponin from *Radix Bupleuri*, was shown to enhance chemosensitivity in intrahepatic cholangiocarcinoma by targeting the p-AKT/BCL-6/ABCA1 axis (49). This aligns with our findings that ginsenoside Rh2 attenuated the LPS-induced inflammatory responses in RAW264.7 macrophages by inhibiting the EGFR/JAK1/STAT3 axis, a critical pathway that drives macrophage activation and enhances cytokine storm. This is aligns with the emerging evidence that STAT3 hyperactivation promotes chronic inflammation in fibrotic liver microenvironments by perpetuating macrophage-epithelial crosstalk and extracellular matrix deposition (50). Notably, the Rh2’s capacity to modulate the pattern recognition receptor signaling and JAK/STAT pathways suggests a polypharmacological mechanism distinct from conventional single-target anti-inflammatory agents. Given its potential to concurrently reduce the production of pro-inflammatory mediators NO, TNF-α, IL-6, IL-1β and elevate regulatory cytokines IL-10 and TGF-β, it can be inferred that it rebalance the macrophage functional states rather than achieve broad immunosuppression. This is particularly relevant in hepatic fibrosis, where excessive STAT3 activation has been shown to exacerbate inflammasome activity and hepatocyte pyroptosis (51).

The observed suppression of IRF5, a master regulator of pro-inflammatory macrophage polarization, provide additional evidence for the role of Rh2 in the reprogramming of transcriptional networks that modulate immune responses. Intriguingly, the Rh2-mediated EGFR inhibition can disrupt a feedforward loop in which, STAT3 activation promotes the EGFR expression, creating a self-reinforcing inflammatory circuit. Spatial transcriptomic evidence generated from *in vivo* models (52) complements the *in vitro* findings, which demonstrates the Rh2’s preferential targeting of periportal macrophages—key drivers of zonal inflammation in fibrotic livers. It capacity to silence Nlrp3 and Asc in these spatially restricted populations suggests that it can also mitigate inflammasome-driven tissue damage while preserving homeostatic macrophage functions in uninjured regions.

Our previous research demonstrated that PZH mitigates fibrosis by inhibiting the TGF-β1/Smad2 signaling pathway, a critical driver of HSC activation and ECM deposition (10). The current study expands upon this mechanistic insight by elucidating PZH’s simultaneous modulation of the EGFR/JAK1/STAT3 signaling axis. Although these pathways predominantly operate within distinct cellular contexts—TGF-β/Smad2 primarily in HSCs and EGFR/JAK1/STAT3 in macrophages—they exhibit significant crosstalk. Notably, TGF-β1 can transactivate EGFR in HSCs through metalloproteinase-dependent shedding of EGFR ligands such as HB-EGF, establishing a feedforward loop that amplifies fibrogenic signaling (53). Furthermore, phosphorylated Smad2/3 and STAT3 can form transcriptional complexes that co-regulate pro-fibrotic genes, including α-SMA and TIMP1 (54). Additionally, macrophage-derived TGF-β1 activates HSCs, while macrophage STAT3 sustains IL-6/gp130 signaling, thereby further enhancing TGF-β responses in HSCs (55). The recent identification of PZH’s modulation of the EGFR/JAK1/STAT3 pathway enhances its known inhibitory effects on the TGF-β1/Smad2 axis, thereby elucidating a multifaceted mechanism against hepatic fibrosis. Future research should focus on delineating the temporal dynamics between these pathways to optimize strategies for combinatorial targeting.

The study shows PZH’s anti-fibrotic effects but has limitations due to its reliance on a CCl_4_-induced mouse model, which limits statistical power and clinical relevance. CCl_4_ models basic fibrosis mechanisms but doesn’t represent major human causes like NAFLD or HBV. While CCl_4_ induces fibrosis through HSC activation and NF-κB inflammation, NAFLD and HBV involve different pathways (56, 57). Thus, validation with human hepatocytes or diverse models is needed. Although no acute toxicity was found in the short term, long-term safety studies are crucial, especially for herb-drug interactions and potential liver issues. Additionally, although ginsenoside Rh2 serves as a significant effector, the synergistic interactions among PZH constituents have yet to be elucidated. Furthermore, while RAW264.7 cells are utilized to investigate conserved signaling pathways, the species-specific nature of immune responses suggests the future application of iPSC-derived macrophages or Kupffer-hepatocyte co-cultures (58).

Our network pharmacology and molecular docking analyses functioned as hypothesis-generating tools intrinsically linked to experimental validation, thereby establishing a computational-to-empirical continuum. For the multi-component PZH formulation, network pharmacology prioritized core targets and identified the enriched EGFR/JAK1/STAT3 pathway from 196 candidate genes. This approach prevented indiscriminate target validation and strategically guided subsequent *in vivo* and *in vitro* experimental designs. Molecular docking further confirmed high-affinity binding of ginsenoside Rh2 to these targets, providing a structural rationale for PZH’s selective pathway modulation. This bridged the mechanistic gap between PZH’s observed anti-fibrotic efficacy and its target specificity, establishing a causal cascade from binding affinity to pathway inhibition and protein downregulation—consistent with prior pharmacological evidence (59). Collectively, these computational methods captured the essence of TCM by elucidating multi-component, multi-target interactions inherent to compound formulae. Critically, all predictions underwent rigorous experimental verification, demonstrating a replicable framework for deconvoluting mechanisms of complex TCM formulations while enhancing methodological rigor. While molecular docking provided valuable insights into the potential binding of ginsenoside Rh2 to EGFR, JAK1, and STAT3, it is important to note that computational affinity predictions do not unequivocally demonstrate functional target modulation within a cellular context. The absence of direct functional validation, such as cellular thermal shift assays (CETSA), surface plasmon resonance (SPR), or co-immunoprecipitation experiments, constitutes a limitation of the current study. Importantly, no direct causal relationship has been established between the molecular docking scores and the observed downregulation of protein expression. The docking results suggest only a potential for binding and do not prove that such binding events directly lead to functional inhibition or changes in the expression levels of the target proteins. Future investigations should incorporate these methodologies to confirm direct target engagement and further elucidate the precise mechanisms by which PZH constituents regulate the EGFR/JAK1/STAT3 signaling axis.

In summary, PZH can exert potent anti-inflammatory and anti-hepatic fibrosis effects due to its multiple bioactive constituents. These components collectively exert anti-fibrotic actions via multi-pathway and multi-target mechanisms, thereby modulating hepatic fibrogenesis. In a CCL_4_-induced mice model, PZH significantly alleviated hepatic inflammation and fibrotic severity, ameliorating liver injury by inhibiting the EGFR/JAK1/STAT3 signaling pathway and pro-inflammatory polarization of hepatic macrophages. This mechanistic interplay highlights the PZH’s capacity to disrupt pathological cascades driving fibrosis progression while preserving hepatic immune homeostasis.

## 5 Conclusion

In brief, the current work showed that PZH may act as a therapeutic agent for liver fibrosis by inhibiting the EGFR/JAK1/STAT3 signaling axis and macrophage M1 polarization. Future research should focus on understanding the roles of PZH components like quercetin and ursolic acid in fibrosis and their interaction with ginsenoside Rh2. Investigating cell-type-specific mechanisms, especially macrophage-hepatocyte interactions, using spatial transcriptomics is essential due to PZH’s targeting of periportal macrophages. Additionally, improving delivery systems, such as nanocarriers, to boost the bioavailability and liver targeting of active ingredients is a key translational goal.

## Data Availability

The raw data supporting the conclusions of this article will be made available by the authors, without undue reservation.
